# Correlate Attitudes Toward LGBT and Sexism in Spanish Psychology Students

**DOI:** 10.3389/fpsyg.2020.02063

**Published:** 2020-08-21

**Authors:** Miguel Ángel López-Sáez, Dau García-Dauder, Ignacio Montero

**Affiliations:** ^1^Departament of Psychology (Social Psychology), Rey Juan Carlos University, Alcorcón, Spain; ^2^Department of Social Psychology and Methodology, Faculty of Psychology, Autonomous University of Madrid, Madrid, Spain

**Keywords:** homonegativity, binegativity, pro-trans, sexism, *ex post* facto study

## Abstract

The present study evaluates the correlations between sexism, homonegativity, binegativity, pro-trans attitudes, political affiliation, contact with LGBT individuals and perceived stigma among psychology students. A study was conducted with 655 cis women (471 heterosexuals, 179 bisexuals and lesbians) and 174 cis men (120 heterosexuals, 54 bisexuals and gays). Descriptive, multivariate analysis of variance, bivariate correlations and multiple regression were used. In general, the groups of men and heterosexuals obtained higher negativity scores and lower acceptance scores, with significant correlations being more frequent in the heterosexual group. Predictive models confirmed the literature on social and ideological conservatism.

## Introduction

One half century after the famous Stonewall uprising in New York City, the violence faced by lesbians (L), gays (G), bisexuals (B), and trans individuals (T) continues to be a social reality. In Spain, one of the countries where LGBT individuals enjoy the greatest number of rights (ILGA, 2019) and where more than 80% of the population supports education on the subjects of gender identity and sexual-emotional diversity (Eurobarometer, 2019), the situation has not always been ideal. For example, in 1975, according to a survey done by the *Revista Guadiana* magazine, 83% of the population believed that homosexuality and everything associated with it (such as trans) should disappear (Mora, 2018). Despite progress, concerns remain about the threat to LGBT rights. In fact, since 2011 Spain has dropped 10 spots to eleventh place due to the absence of legislation that guarantees protection (ILGA, 2019). According to the Eurobarometer (2019), the low perception of discrimination held by the population (between 39 and 54% according to the area) contrasts with an increase in hate crime incidents: from 169 in 2015 to 271 in 2017 (Ministry of the Interior, 2018); or from 107 in 2015 (Martín-Peréz et al., 2016) to 623 in 2017 (Rebollo et al., 2018).

There are no clear numbers about incidents in the university context, although some studies have cautioned that Spanish universities are not violence-free areas (FELGTB, 2013; Rebollo et al., 2018). Incidents involving Spanish professors (Borraz, 2018; Peñalver, 2018) and campaigns carried out by organizations opposed to educational coexistence and inclusion (Arribas, 2019) serve as more general indicators of these systems of violence.

Because of the possibility of differentiating hate crimes or incidents against the LGBT community from others, the motivation can be deduced: LGBTphobia, a motivation based on a set of beliefs that result in abuse and discrimination. An incident is the more visible consequence of imperceptible attitudes. The study of attitudes, however, has received less attention. While institutions focus on consequences that expose an urgent social problem (Cvetkovich, 2018), there are other forms of hate that do not manifest themselves in the form of an incident or crime: the expression of negative attitudes.

The discourses of the various social and health sciences have also been imbued with these negative attitudes, in particular psychology. Inside this discipline, the clinical branch monopolized the study of working with LGBT individuals. In Spain, the discipline maintained a pathologizing discourse until the mid-1980s (Mora, 2018). However, an international evolution, guided by American psychology, had several phases. Beginning in the 1930s and until the 1970s, within the paradigm of differences in personality features, a wide range of theories and tools were developed to justify the inferiority of women and to detect and/or correct deviations in gender expression, orientation and identity (López-Sáez et al., 2019). It was not until 1975 that the American Psychological Association supported eliminating homosexuality as a disorder, one year later than its psychiatric colleagues working on the DSM-II. Four years later, the same situation occurred with transsexuality in the DSM-III. While in theory, homosexuality and transsexuality officially disappeared as disorders, in reality, they continued to be pathologized in other categories (Grau, 2017). The evolution of the two “sick-making” processes (homosexuality and trans) followed the same course in that the malaise was attributed to not following the cisheteronormative patterns used as diagnostic indicators of identity disorders. Moreover, until quite recently, Spanish psychologists continued to employ instruments with items whose contents were used to “diagnose” this split (Marano, 2009).

However, as observed in the recent literature on the subject (Meyer, 2003; López-Sáez et al., 2019), thanks to criticism from social psychology and other disciplines, some transformations took place. The perspective of the pathologizing diagnosis that placed responsibility on the transgressor of the norm (traditional, heterosexual and cisgender femininity/masculinity) using tools to evaluate personality features was replaced by one that problematized those who penalized the transgressions using evaluations. The scientific literature has described these attitudes as phobias or negativities. Although some authors currently defend the use of an umbrella term like “sexual prejudice” (Herek and McLemore, 2013), this study prefers to recognize differentiation, linkages and the idiosyncrasies of different LGBT individuals.

The order in which the studies of each negativity regarding dissident identities emerged is not arbitrary in a scientific tradition that has focused on male homosexuality. The tradition of measuring homonegativity is more extensive when compared to binegativity or transnegativity. Initially, consistent with Alfred Kinsey’s polarized classifications and Michael Storms’s dimensional focus, works concentrated on looking at phobia toward gays and lesbians (Smith, 1971; Weinberg, 1972).

The term “phobia” in and of itself has been the object of theoretical discussion, since the fear and apprehension linked to its meaning limits the inclusion of a wide range of negative connotative inferences, cognitions and feelings (Hudson and Ricketts, 1980; Fyfe, 1983; Haaga, 1991; Logan, 1996). Given this, a proposal was made to use “homonegativity” (Hudson and Ricketts, 1980) to define the valence of the type of attribution, and “heterosexism” (Herek, 1992; Nakayama, 1998) to reveal the place of heterosexual privilege. However, the term that continues to be most often used on a colloquial basis is “homophobia” (Borrillo, 2001). On the basis of the given definitions, homonegativity can be summarized as attitudes prejudiced against gay-lesbian identity or anything associated with it. “Anything associated with it” is understood to refer to deviations from the coherence between gender expression, erotic desire and assigned gender identity. Therefore, homonegativity is not only experienced by LG, but also by other individuals whose deviation is associated with homosexuality, for example, an effeminate man or a masculine woman (Guash, 2006).

Some studies have argued that socialization in heterosexual masculinity is in and of itself homophobia, since it views male femininity as a threat (Worthington et al., 2002; Warriner et al., 2013). In that respect, a number of previous studies have found connections between those who inhabit a gender identity as men, sexism, homophobia and conservatism (Warriner et al., 2013; Worthen, 2013; Dierckx et al., 2017; Rye et al., 2019).

Looking back, the first tools that measured homonegativity – although this was not their objective – were the masculinity scales of the 1930s (López-Sáez and García-Dauder, 2020). However, the first instruments explicitly created for that purpose began to appear in the 1970s, when the paradigm shifted and specific tools to detect manifest negative attitudes were developed (Hudson and Ricketts, 1980; Aguero et al., 1984). During the 1990s and beyond, the emphasis was placed on detecting more subtle beliefs (O’Donohue and Caselles, 1993; Wright et al., 1999). Currently, the academic debate is focusing on searching for theories and instruments to provide information that can detect more imperceptible and modern forms of homonegativity. According to Morrison and Morrison (2002), one of the most often cited works, some of the most prominent beliefs that characterize these less visible forms argue that: the question of inequality is out-dated, rarely encountered today and is not serious; demands for rights are exaggerated and radical; and homosexuality is tolerable, as long as its expressions are not aired. In addition, the results found by Morrison and Morrison (2002) showed higher scores for heterosexual men than for women and support the presence of correlations among political conservatism, sexism, and homonegativity.

Recent works have found additional connections on which new beliefs are based: sexist perspectives that establish spheres of masculinity and femininity are related to considerations regarding the supposed suitability of gays and lesbians for specific stereotyped roles (Walls, 2008); being part of cisheterosexual privilege correlates with disinterest (willful ignorance) of LGBT realities (Brownfield et al., 2018); and the false respectability of any orientation, as long as it is kept outside the family (Walls, 2008).

The interest in attitudes toward bisexuals has been much more recent in the empirical literature (Mulick and Wright, 2002), despite their being a majority in the LGBT community (Copen et al., 2016), having worse mental and physical health (Shearer et al., 2016) and being more affected by stressors like abuse and cyberabuse (Kann et al., 2018). The term “biphobia” did not appear until 1992 (Bennett, 1992) and although the authors of the most widely used scale were still using it in 2008 (Mulick and Wright, 2002), in the 2012 revision, Yost and Thomas recommended using “binegativity,” for reasons similar to those surrounding the use of “homonegativity.” The delay was due to the categorization of biphobia as part of homophobia. This explanation is based on the idea that desire for the same gender identity is read as transgressing the heterosexual norm. However, although homophobia and biphobia share some roots because of the heteronormative split, bisexuals also break from the monosexuality implied by gay-lesbian desire. Biphobia is the result of a dual delimited dichotomy between heterosexual-homosexual and men-women (Hertlein et al., 2016). Bisexual invisibility is a consequence of the belief that defines bisexuality as an invalid or unreal orientation (Burke and LaFrance, 2016), confusion or indecision (Dyar et al., 2017) or transition (Alarie and Gaudet, 2013) in the best of cases. The first scales that approached binegativity as a separate entity outside the umbrella of homosexuality focused on the attitudes of the heterosexual population (Eliason, 1997) and later included gays and lesbians (Mulick and Wright, 2002). Mulick and Wright (2002) were the first to highlight the importance of sexual orientation in measuring biphobia, raising the possibility that negative attitudes come not only from heterosexuals, but also from gays and lesbians. Their results showed a higher level of biphobia among heterosexuals, correlations among homophobia, biphobia and conservative beliefs, and a higher intensity of correlations in the heterosexual sample. Current studies continue to investigate the nuances of sexual orientation in the negative assessment of bisexuality: from the heterosexual perspective, bisexual women/men are “lacking in real desire”/“embarrassing to manhood,” and from the lesbian-gay point of view, they are “traitors and heterosexuals”/“not daring to face stigma and true gays” (Matsick and Conley, 2016; Matsick and Rubin, 2018). Yost and Thomas (2012), in turn, highlight the importance of considering gender identity as a key variable for a better understanding of the construct of binegativity. Their results indicate greater binegativity against bisexual men among heterosexual men.

Alongside these deviations from the paths that guide desire toward heterosexuality and shape correct gender expressions (Ahmed, 2019), others have emerged that question the medical identity assigned at birth (Fausto-Sterling, 2006). These trans and gender diverse (fluid or non-conformist) individuals experience more violence, as different axes of oppression, such as sexism, homophobia and the like, intersect in them. This was highlighted by data in the report “Being Trans in the EU” (FRA, 2014), which found that trans individuals experience greater discrimination in the workplace, education and healthcare, among other areas. They are subjected to violence that depends on performative success according to cisheteronorms (CIDH, 2015).

The amount of violence contrasts with the lack of systematic research into the attitudes that generate it (Hill and Willoughby, 2005; Kanamori et al., 2017). Nonetheless, the first studies of this phenomenon appeared a decade before studies on homonegativity. Initial interest in measuring transphobia can be explained by theories on the subject of the paradigm of gender identity developed by Robert Stoller and John Money in the 1950s and 60s. The first instrument sought to explore “negative attitudes toward trans” among health professionals (Green et al., 1966). However, it was not until 2002 that Hill used the term “transphobia,” conceptualized as hatred or emotional repugnance toward those who do not meet the cisheteronorm imposed by a stereotyped dualist ideology (Hill, 2002). In the same vein, other studies refined the measurements and components that form part of transphobia. Furthermore, these studies brought clarity to the connections between the prejudices that share roots in terms of gender transgression (whether expression, roles or sexuality), as shown by the positive and significant correlations among transphobia, LGBnegativity, sexism (Hill and Willoughby, 2005; Tebbe et al., 2014) and political conservatism (Nagoshi et al., 2008). Additionally, according to Nagoshi et al. (2008), gender identity is an important variable that differentiates men from women in that women were less transferable and their transphobia could be predicted on the basis of benevolent sexism.

In 2017, Kanamori et al. (2017) preferred to speak of “attitudes toward trans” and added culturally important elements like advances in civil rights and beliefs associated with biology and conservative and religious moralities. In this way, they proposed an approach focused more on the acceptance of trans people and less on negativity. Their results indicated that the gender identity of the evaluators makes a difference, with men scoring lowest in trans acceptance on all dimensions.

As seen above, various negative beliefs pervade the narrative about LGBT in the form of emotional inferences and attributions and conduct. Through them, an amalgam of explicit and/or subtle rejections are reflected that comprise ambivalence patterns that change over time. This structure is similar to the construct of sexism, where different models have posited a more modern, subtle, benevolent bidimensionality on the one hand, and a more traditional, explicit and hostile one on the other (Glick and Fiske, 1997). Sexism is defined by beliefs that justify the supremacy of the male over the female; delimit the roles, characteristics and behaviors suitable for men and women; establish heterosexuality as obligatory and necessary for the maintenance of power; and consider any opportunity for equality to be excessive and unnecessary. Consequently, the analogy between sexism and other constructs seems to comply with an ideology that establishes situations of subordination and subjugation for those who depart from the prescribed course.

Therefore, it is no surprise that, as with sexism, male subjects tend to score higher and correlate positively with the different constructs that define negative attitudes toward LGBT people. In other words, based on previous literature, gender identity has been a key variable in determining the levels of LGBTnegativity and sexism. However, this is not the only variable, since sexual orientation intersects with gender identity, configuring normality and queer. LGBT people may display negative attitudes toward any of the acronyms in their group and even against their own, due to an internalization of cisheteronorms. However, few studies have advanced in fully understanding attitudes toward LGBT individuals (with a measurement that takes into account more than one or two of the constructs seen) and the importance of the intersection of gender and sexual orientation (Worthen, 2013; Tebbe et al., 2014).

All of the scales and theories about negativities share connections, being based on conservative ideological and sexist concepts. A large number of studies view political conservatism and sexism as predictor variables when it comes to anticipating anti-LGBT attitudes (Warriner et al., 2013; Austin and Jackson, 2019). Other studies indicate a lack of contact with LGBT individuals as a variable that is closely connected with levels of negativity (Lytle and Levy, 2015; Badenes-Ribera et al., 2016; Dierckx et al., 2017).

The aim of this study, carried out with heterosexual and LGB individuals, is to promote an understanding of the correlations among each of the constructs examined above, as well as their connection to sexism, political conservatism, contact networks and perceived or experienced stigmatization. The analysis is situated in the current Spanish context where, despite advances, social, professional and academic arguments in the field of psychology continue to be made that endorse discriminatory positions (De Benito, 2005; Ruíz, 2019; Villascusa, 2019). Furthermore, studies of attitudes toward LGBT individuals in the context of education have barely mapped the Spanish university situation (Penna, 2012; Varo et al., 2015) and even less so in the field of psychology. In the Spanish context, it is noteworthy that there are no compulsory classes on gender, diversity and health in the psychology degree, particularly given that the basic degree allows graduates to practice professionally as psychologists with all the legal competencies and functions of the profession. Therefore, and more than ever, the recommendations found in the APA (2012, 2015) guidelines and other organizations are relevant. They call for a review of the attitudes of future generations of professional psychologists (Kite and Bryant-Lees, 2016). We argue that have an understanding of the collective thinking regarding sex and gender diversity and better comprehending the relationships between negativities and the factors that underpin them, is essential for an initial screening of the situation in Spain.

## Materials and Methods

### Participants

A representative sample of 831 students from three public universities in Madrid who are studying psychology took part. Regarding the academic year, 50% belonged to the first cycle (first and second academic years) and 50% to the second cycle (third and fourth academic years).

### Instruments

Except for the questionnaire on sociodemographic aspects, the scales used a response format from 1 (strongly disagree) to 6 (strongly agree) in order to avoid neutral answer trends and to homogenize the survey information.

#### Sociodemographic Questionnaire

This included gender identity, sexual orientation, age, academic year, nationality, socioeconomic level, political affiliation, contact and perceived stigma.

For gender identity, although the student sample had originally been selected on the basis of the data provided to each university, the question was asked again to avoid any inaccuracies. The self-report provided several closed options (cis man, cis woman, trans man, trans woman, fluid gender, non-binary gender) and one open option that could be filled in (for people who did not choose from one the above categories).

For sexual orientation, like gender identity, several closed categories were offered (heterosexual, gay-lesbian, bisexual, asexual, pansexual, demisexual) and one open one that could be filled in.

For the academic year (1st, 2nd, 3rd, 4th) and for nationality, closed categories were offered with the full range of options. For age, participants wrote down the number that corresponded to them.

For political affiliation, a single-element measurement was used (Gerbner et al., 1984), based on a 4-point Likert scale (left = 1, center-left = 2, center-right = 3, right = 4) and the political affiliation variable appears as “political conservatism or right-wing political”.

For contact, three items were used that asked about the existence or lack of contact with LG, B, and T individuals in some social circle. These items used a dichotomous response format (yes = 1, no = 2) and the contact variable appears as “lack of contact or no contact”.

Perceived stigma was determined by the question used by Hertlein et al. (2016): “Is it ever easier or preferable to not self-identify your sexual orientation in certain situations or with certain people?” The response option was dichotomous (yes = 1, no = 2).

#### Ambivalent Sexism Inventory (ASI)

In its short version, this consists of 12 items to evaluate sexism using two subscales that measure hostile sexism (ASI-HS) and benevolent sexism (ASI-BS). Rollero et al. (2014) report a good alpha coefficient of internal consistency (ASI-HS, α = 0.85; ASI-BS, α = 0.80). In this study, the internal consistencies were 0.84 and 0.70, respectively.

#### Modern Homonegativity Scale (MHS)

This 24-item scale measures contemporary negative attitudes toward gays and lesbians. The items “gay men/lesbian women who are ‘out of the closet’ should be admired for their courage” were found to be particularly ambiguous and were, thus, eliminated. The highest scores indicate greater contemporary negativity. Morrison and Morrison (2002) report alpha coefficients with optimal internal consistency for gays (α = 0.91) and lesbians (α = 0.87). In this study, the internal consistencies were 0.87 and 0.88, respectively.

#### Biphobia Scale (BphS)

This scale consists of 30 items that provide a measurement of negative attitudes toward bisexuality. Despite including items that touch on cognitive, emotional and behavioral factors, it is unidimensional. The higher scores indicate greater binegativity. Mulick and Wright (2002) report an alpha coefficient of internal consistency of 0.94. In this study, the internal consistency was 0.81.

#### Transgender Attitude and Belief Scale (TABS)

This 29-item scale measures attitudes toward trans individuals through three dimensions: interpersonal comfort; beliefs regarding gender identity; and human value. High scores indicate a greater degree of acceptance of trans diversity. The items were adapted, replacing the term “transgender” with “trans,” since that picks up on a greater variety of identities and gender expressions that diverge from the gender medically that was assigned at birth (for example: transgender, transsexual, gender fluid or non-binary, queer, etc.) Kanamori et al. (2017) report an optimal alpha coefficient of internal consistency (α = 0.98). In this study, the internal consistency was 0.88.

### Procedure

A stratified random sampling was used with proportional allocation for each of the three universities. Out of a total population of 3,745 students, the sample size was determined for a confidence level of 95%, a maximum variability and a maximum error of ±3%. The groups for each level were selected randomly. The selection of participants followed proportional criteria according to gender identity (men, women) and the academic year recorded in the academic records of each university. The rejection rate of the selected individuals was 30%. At two of the universities, the selected individuals were contacted when attending one of their face-to-face classes. At another university, people were contacted by email. In any case, all participants accessed an online questionnaire. All the participants received the same instructions and were informed that their participation was voluntary and their responses confidential. Before beginning, they had to read and accept the informed consent. The study was approved by the Autónoma University Research Ethics Committee, which coordinated the study.

## Results

### Descriptive Statistics

In total, 79% of the participants were cis women and 21% cis men. Due to the low sample size (*N* = 2), trans students were excluded from the analysis for this study. Among the cis women, 72.7% identified themselves as heterosexual, 25.8% as bisexual and 1.5% as lesbian. Among the cis men, 69% identified themselves as heterosexual, 13.8% as bisexual and 17.2% as gay. The ages ranged from 17 to 60 (asymmetry = 6.09, Mdn = 20, Mo = 19). Almost all the participants self-identified as middle-lower class (36.1%) or middle-upper class (57.3%), while very few considered themselves either lower class (4.7%) or upper class (1.9%). With regard to political affiliation, 42.2% identified with the left, 35.7% with the centre-left, 19.5% with the centre-right and 2.5% with the right.

Due to the small sample size of gay (*N* = 30) and lesbian (*N* = 10) individuals, when segmenting by gender identity, they were grouped with the bisexuals, leaving one LGB group (*N* = 233) and one heterosexual group (*N* = 596). Additionally, the homonegativity scores toward gays and homonegativity scores toward lesbians were averaged to obtain a single score in order to prevent problems of collinearity and to be able to make comparisons with the other scales that did not provide a specific negativity according to gender identity.

Descriptive statistics were obtained for each variable, along with a visual examination of histogram and normality tests. The scores for each dimension were calculated by averaging the items.

Table 1 presents the means and standard deviations by gender identity (men/women) and sexual orientation (heterosexuals/LGB) for MHS, BphS, TABS, ASI, political conservatism, lack of contact, and perceived stigma.

**TABLE 1:** Means and standard deviations by gender identity and sexual orientation.

	Heterosexuals				LGB			
		
	Men		Women		Men		Women	
				
	*M*	*SD*	*M*	*SD*	*M*	*SD*	*M*	*SD*
Homonegativity (MHS)	2.38	1.04	1.91	0.75	1.52	0.60	1.38	0.40
Binegativity (BphS)	1.27	0.39	1.16	0.22	1.16	0.21	1.07	0.10
Pro-trans (TABS)	5.37	0.68	5.64	0.42	5.65	0.46	5.79	0.22
Hostile sexism (ASI-HS)	2.15	0.99	1.53	0.63	1.39	0.54	1.30	0.50
Benevolent sexism (ASI-BS)	2.26	0.98	1.98	0.70	1.99	0.71	1.76	0.60
Right-wing political	2.04	0.87	1.97	0.83	1.43	0.63	1.42	0.65
No LG contact	1.23	0.43	1.09	0.29	1.04	0.19	1.04	0.21
No B contact	1.27	0.45	1.20	0.40	1.06	0.23	1.04	0.21
No T contact	1.93	0.25	1.82	0.39	1.50	0.51	1.61	0.49
Perceived stigma	0.03	0.18	0.07	0.25	0.83	0.38	0.82	0.39

### Multivariate Analysis of Variance

Differences in gender identity and sexual orientation were analyzed using a multivariate analysis of variance (MANOVA). The scales related to political conservatism, lack of contact and perceived stigma were considered dependant variables, while gender identity and sexual orientation were the independent variables.

The results from the MANOVA indicated significances in the interaction (gender identity/sexual orientation) *F*(10, 816) = 3.64, *p* < 0.001, η*_*p*_*^2^ = 0.04.

In MHS, the interaction between gender identity and sexual orientation was significant [*F*(1, 825) = 5.50, *p* < 0.05, η*_*p*_*^2^ = 0.007]. This type of interaction requires simple-effects analyses to be interpreted without error (see León and Montero, 2015). The simple-effects analyses for gender identity showed that significant differences existed between heterosexual men and women [*F*(1, 594) = 21.09, *p* < 0.001, η*_*p*_*^2^ = 0.05] and LGB [*F*(1, 231) = 4.45, *p* < 0.05, η*_*p*_*^2^ = 0.02], with men scoring higher. The simple-effects analyses for sexual orientation showed that for both women [*F*(1, 653) = 80.58, *p* < 0.001, η*_*p*_*^2^ = 0.11] and men [*F*(1, 172) = 31.32, *p* < 0.001, η*_*p*_*^2^ = 0.15], heterosexuals scored higher than LGB for homonegativity.

In ASI-HS, the interaction between gender identity and sexual orientation was significant [*F*(1, 825) = 18.01, *p* < 0.001, η*_*p*_*^2^ = 0.02]. The simple-effects analyses for gender identity showed significant differences between heterosexual men and women [*F*(1, 594) = 70.88, *p* < 0.001, η*_*p*_*^2^ = 0.11], with the men scoring higher. However, among LGB individuals [*F*(1, 231) = 1.34, *p* = 0.25, η*_*p*_*^2^ = 0.006] there were no significant differences. The simple-effects analyses for sexual orientation showed that for both women [*F*(1, 653) = 20.42, *p* < 0.001, η*_*p*_*^2^ = 0.03] and men [*F*(1, 172) = 28.03, *p* < 0.001, η*_*p*_*^2^ = 0.14], heterosexuals scored higher than LGB for this type of sexism.

The interactions between gender identity and sexual orientation were also significant regarding lack of LG contact [*F*(1, 825) = 7.14, *p* < 0.01, η*_*p*_*^2^ = 0.009] and T contact [*F*(1, 825) = 8.86, *p* < 0.01, η*_*p*_*^2^ = 0.011]. The simple-effects analyses for gender identity showed significant differences between heterosexual men and women in the lack of LG contacts [*F*(1, 594) = 17.63, *p* < 0.001, η*_*p*_*^2^ = 0.03] and T contacts [*F*(1, 594) = 9.50, *p* < 0.01, η*_*p*_*^2^ = 0.02], with men scoring higher. However, there were no significant differences among LGB individuals [LG, *F*(1, 231) = 0.06, *p* = 0.81, η*_*p*_*^2^ = 0.0002; T, *F*(1, 231) = 2.03 *p* = 0.16, η*_*p*_*^2^ = 0.009]. The simple-effects analyses for sexual orientation showed significant differences among both women [LG, *F*(1, 653) = 4.36, *p* < 0.05, η*_*p*_*^2^ = 0.007; T, *F*(1, 653) = 33.25, *p* < 0.001, η*_*p*_*^2^ = 0.05] and men [LG, *F*(1, 172) = 10.55, *p* < 0.001, η*_*p*_*^2^ = 0.06; T, *F*(1, 172) = 57.37, *p* < 0.001, η*_*p*_*^2^ = 0.25], with heterosexuals scoring higher.

In ASI-BS, BphS and TABS, both gender identity and sexual orientation showed significant primary effects. The primary-effects analyses for gender identity in ASI-BS [*F*(1, 825) = 14.51, *p* < 0.001, η*_*p*_*^2^ = 0.02] and BphS [*F*(1, 825) = 22.83, *p* < 0.001, η*_*p*_*^2^ = 0.03] showed significant differences between men and women, with men scoring higher. The primary-effects analyses for sexual orientation showed significant differences between heterosexuals and LGB individuals [ASI-BS, *F*(1, 825) = 13.18, *p* < 0.001, η*_*p*_*^2^ = 0.02); BphS, *F*(1, 825) = 19.22, *p* < 0.001, η*_*p*_*^2^ = 0.02], with heterosexuals scoring higher. Similarly, but conversely, in TABS, the primary-effects analyses for gender identity [*F*(1, 825) = 25.01, *p* < 0.001, η*_*p*_*^2^ = 0.03] and sexual orientation [*F*(1, 825) = 27.38, *p* < 0.001 η*_*p*_*^2^ = 0.03] showed significant differences between men and women and between heterosexuals and LGB individuals, with women in both groups and LGB individuals scoring higher.

Finally, the primary-effects analyses for sexual orientation in political conservatism [*F*(1, 825) = 62.50, *p* < 0.001, η*_*p*_*^2^ = 0.07] and lack of B contact [*F*(1, 825) = 30.13, *p* < 0.001, η*_*p*_*^2^ = 0.04] showed significant differences between heterosexuals and LGB, with heterosexuals scoring higher. Conversely, in the primary-effects analysis for sexual orientation in perceived stigma [*F*(1, 825) = 853.71, *p* < 0.001, η*_*p*_*^2^ = 0.51], LGB individuals scored higher.

### Correlations and Multiple Regression Analysis

A bivariate correlation analysis was done using the same variables. The correlations were estimated using Spearman’s ρ coefficient due to the violation of the assumptions of continuity or normality in all of the pairs of variables. Table 2 shows the correlations for MHS, BphS, and TABS with themselves and with ASI-BS and ASI-HS, political conservatism and perception of stigma for heterosexual men and women and LGB individuals.

**TABLE 2:** Correlations and correlations by gender identity and sexual orientation.

	Heterosexuals			LGB		
		
	Homonegativity (MHS)	Binegativity (BphS)	Pro-trans (TABS)	Homonegativity (MHS)	Binegativity (BphS)	Pro-trans (TABS)
**Men**						
Binegativity (BphS)	0.46**			0.50**		
Pro-trans (TABS)	−0.59**	−0.63**		−0.54**	−0.46**	
Hostile sexism (ASI-HS)	0.72**	0.41**	−0.52**	0.48**	0.24	−0.36**
Benevolent sexism (ASI-BS)	0.31**	0.30**	−0.34**	0.34*	0.01	–0.24
Right-wing political	0.50**	0.27**	−0.40**	0.21	–0.05	–0.22
No LG contact	0.23*	0.27**	−0.36**	0.05	–0.04	–0.10
No B contact	0.25**	0.32**	−0.27**	0.03	0.15	–0.05
No T contact	0.05	0.06	–0.17	0.17	–0.17	–0.08
Perceived stigma	–0.03	–0.01	0.01	–0.13	–0.09	0.17
**Women**						
Binegativity (BphS)	0.41**			0.20**		
Pro-trans (TABS)	−0.54**	−0.43**		−0.17*	−0.28**	
Hostile sexism (ASI-HS)	0.57**	0.29**	−0.35**	0.47**	0.12	–0.07
Benevolent sexism (ASI-BS)	0.42**	0.24**	−0.27**	0.19*	0.06	–0.10
Right-wing political	0.50**	0.28**	−0.38**	0.27**	0.12	0.18
No LG contact	0.03	0.11*	–0.06	0.06	0.17*	–0.04
No B contact	0.18**	0.27**	−0.25**	0.10	< −0.01	–0.10
No T contact	0.11*	0.01	−0.15**	0.07	–0.02	–0.07
Perceived stigma	−0.13**	–0.01	0.07	−0.27**	−0.21**	0.26**

*p < 0.05; **p < 0.01.

Additionally, a multiple regression analysis was carried out using the stepwise method. The model analysis was done separately for four groups according to gender identity and orientation. Moreover, and according to the pertinent literature, homo-binegativity (MHS and BphS) and pro-trans attitudes (TABS) were predicted based on sexism (ASI-BS and ASI-HS) and political conservatism. The predictive potential of the models was done using the adjusted *R*^2^ statistic, while the predictive potential of each predictor was evaluated using its standardized dependent variable with confidence intervals of 95%. The assumptions were evaluated using collinearity statistics, Q-Q and residual plots and the Durbin-Watson statistic.

The results in Table 3 present the analyses of the model for men and women for the MHS, BphS, and TABS predictions.

**TABLE 3:** Multiple regression in terms of sexism and political conservatism.

	Homonegativity (MHS)			Binegativity (BphS)			Pro-trans (TABS)		
			
	βa	R^2^	Change	βa	R^2^	Change	βa	R^2^	Change
**Heterosexuals**									
*Men*									
Hostile sexism (ASI-HS)	0.61	0.52^a^	0.52***	0.35	0.35^b^	0.11***	-0.33	0.29^a^	0.29***
Benevolent sexism (ASI-BS)	–	–	–	0.37	0.25^a^	0.25***	-0.24	0.34^b^	0.06**
Right-wing political	0.24	0.57^b^	0.04**	–	–	–	-0.23	0.38^c^	0.04**
*Women*									
Hostile sexism (ASI-HS)	0.37	0.31^a^	0.31***	0.14	0.17^c^	0.02**	-0.20	0.20^b^	0.05***
Benevolent sexism (ASI-BS)	0.16	0.43^c^	0.02***	0.21	0.11^a^	0.11***	-0.12	0.21^c^	0.01*
Right-wing political	0.32	0.41^b^	0.10***	0.19	0.16^b^	0.04***	-0.27	0.15^a^	0.15***
**LGB**									
*Men*									
Hostile sexism (ASI-HS)	0.49	0.28^a^	0.28***	–	–	–	-0.33	0.32^b^	0.11**
Benevolent sexism (ASI-BS)	0.26	0.34^b^	0.06*	–	–	–	-0.42	0.21^a^	0.21***
*Women*									
Hostile sexism (ASI-HS)	0.40	0.28	0.28***	0.37	0.14	0.14***	–	–	–

*p < 0.05; **p < 0.01; ***p < 0.001. ^*a*^First predictor. ^*b*^Second predictor adding the previous one. ^*c*^Third predictor adding previous ones.

As the tolerances for all the variables introduced were above 0.10, multicollinearity between the predictors was discarded.

Among the heterosexual women, both sexism (ASI-HS and ASI-BS) and political conservatism were predictor variables on the whole for MHS and BphS and negatively for TABS. On the contrary, among LB women, only ASI-HS was a predictor for MHS and BphS (since TABS had no predictors).

Among men, the components of the predictor models were more heterogenous. Among heterosexual men, ASI-HS and political conservatism were positive predictors for MHS, and both sexisms (ASI-HS and ASI-BS) for BphS, while both sexisms and political conservatism were negative predictors for TABS. Among GB, both sexisms positively predicted MHS and negatively predicted TABS. All the determination coefficients were above 0.35, with most of the cases having moderate to high level predictions.

## Discussion

This study has made it possible to explore the correlations between constructs and analyze their behavior and the differences between heterosexual men and women and LGB individuals. It also contributes to the theoretical postulates regarding predictor variables related to LGBT-negativity.

The results affirm that sexual orientation is a determinant variable with regard to the degree of LGBT-negativity. In this respect, LGB individuals show less sociopolitical conservatism (with a political affiliation inclined toward the left and fewer sexist beliefs) and less homo-binegativity, as well as attitudes that are more favorable toward trans individuals, more contacts with LGBT individuals and a lower perception of stigma. Moreover, regarding MHS, ASI-HS and the lack of LG and T contacts, gender identity indicated significant differences. While the differences between heterosexual men and women were greater, for LGB men and women, they were not. However, being a man or woman was significant among LGB individuals. One sign of this was the higher level of ASI-BS among GB men. This is consistent with the earlier literature that defines masculinity by the negative assessment of any otherness associated with feminity (Worthington et al., 2002; Warriner et al., 2013; Ahmed, 2019). Despite forming part of a group that is subject to violence, the pyramid of privilege that demarcates sexist spheres allows for stigmatization and the negative assessment of those left behind (Glick et al., 2015). The importance of gender identity is also significant in the differences in BphS and TABS, which seems to indicate a stagnant, binary perspective on the part of men.

The correlational findings confirm the relationship between the different constructs, with ASI-HS having the most significant correlations with the other variables. Additionally, the correlations between TABS and BphS were high, which could be consistent because of the proximity between constructs that posit non-monosexual or non-monoidentity possibilities.

In the heterosexual sample, all the variables had significant correlations with each other, except for the lack of contact and perceived stigma. The correlations regarding the lack of contact were uneven between the men and women. For both groups, the lack of B contacts correlates positively with MHS and BphS, but negatively with TABS. The lack of LG contacts has these correlations in the case of men, while for women the lack of T contacts was significant. These differences between heterosexual men and women are consistent with models of identity construction and socialization. Accepting gender identity as a stagnant, biological category generates perspectives that promote heteronormativity and are alert to any performativity. In this way, heterosexual men avoid any proximity or association with “gay influence” (Goldstein and Davis, 2010). In addition, as guardians of gender essence, some women reject trans pronouncements that alter biology (Butler, 2017).

In the LGB population, the correlational differences between men and women had different connection patterns. For men, sexisms correlated significantly with MHS and TABS, while for women, only ASI-BS did so with MHS. One explanation for this is that in GB men, sexist beliefs prevent any confusion with women. As GB men have been socialized to reject effeminacy, their “homoerotic desire” must be homonormativized without this entailing any loss of privileges (López-Sáez, 2017). However, according to the findings by Warriner et al. (2013), LB women may assess the loss of status that comes with heterosexuality as more threatening than a change in gender identity.

Comparing the heterosexual and LGB samples, there are no significant correlations between sexisms and BphS among LGB individuals. Among heterosexual and LB women, for the former, sexisms correlated with all the constructs (MHS, BphS and TABS), while for the latter, sexisms only significantly correlated with MHS. These correlations may be due to the threatening self-perception of the status that LB women have of their own sexuality. This is even more true for LB women with conservative political beliefs, whose values argue for maintaining the traditional spheres of masculinity and femininity in line with particular expressions of desire. In this respect, there was a correlation between political conservatism and MHS. In contrast, the diffusion of correlations between political conservatism and BphS and TABS may be related to mythologized beliefs that view bisexuality and trans as “partial” or “temporary” breaks.

However, this is not the case for conservative heterosexual women, who appear to consider bisexuality and trans as not part of “the right thing.” These differences between LB and heterosexual women help to explain how orientation and gender identity intersect in perceived stigma. As observed by Brownfield et al. (2018), heterosexuality constitutes subjects of privilege who generate stigma of which they are not aware. In contrast, LB women do perceive and experience this stigma, and they engage in it less (less LGBT-negativity).

Comparing heterosexual men with GB men, political conservatism loses significance with all the constructs. This seems to indicate that among heterosexual men, conservative belief systems are more determinant when assessing LGBT as a threat. As Warriner et al. (2013) explain, sharing conservative political beliefs lays down certain guidelines about the role that men should play that prevent any deviation from cisgender and heterosexuality.

The regression analysis provides important information to add to the correlations. Confirming the earlier literature, models that combine sexist and political conservatism are predictors of LGBT-negativities. This is seen among heterosexual women and, with some variations, among heterosexual men, where components of the model alternate. In the LGB sample, political conservatism lost its predictive potential. One explanation for this is that LGB individuals show less political conservatism and less variability in their political affiliation. Finally, the lack of predictors for TABS and BphS may be related to mythologized beliefs about the partial or temporary nature of the situation that does not require a complete break with having sexist and conservative values.

## Conclusion

In conclusion, this study serves as a first step in exploring these constructs among psychology students in the Spanish context. It helps to better explain the complexities of the beliefs that underpin LGBTphobic discrimination, observing the differences according to sexual orientation and gender identity. However, it is only the beginning of a long road that must accept intersectionality as the essential foundation for its development. For that reason, more research is required to help explain the complex articulations of LGBT-negativity. Future studies should also expand the size of the LGB sample in order to explore the differences with bisexuals in detail. It would also be interesting to develop longitudinal studies that could include a retrospective perspective as knowledge of psychology is acquired. Moreover, despite having carried out a probability sampling with a considerable sample size, the results are only representative of the public university system. Additionally, the population of psychology students is clearly feminized, represents mid-to-high socioeconomic levels and shares ideological patterns that are not very conservative. These patterns may be different in the case of private secular and religious universities. Similarly, the low racial mix, with a predominance of Caucasians, may have had an influence on certain nuances. Future research should therefore explore the connections established in more heterogeneous probability samples.

Finally, with regard to practical consequences, we must consider how negative attitudes toward LGBT people signal less competence in every type of psychological intervention or accompaniment. This is not only true in the work done with the LGBT population itself, but with the general population. The APA (2012, 2015) guidelines have been scrupulously clear in this respect, indicating the importance of training in the LGBT field for personal review and the recognition of privileges and prejudices. The Spanish National Agency (ANECA, 2005) has asserted that it is essential for psychology students (particularly certain profiles) to receive training in sexual-emotional diversity, specifically, and to fully understand human diversity in general in order to ensure equal opportunities and non-discrimination.

National and international guidelines suggest that the use of novel approaches in the evaluation of training programs in psychology may significantly assist in detecting deficits. Analyses and studies that address attitudes toward sexual orientations and gender identities will make it possible to map educational processes and materials for psychology students and implement changes in the curricula.

## Data Availability Statement

The raw data supporting the conclusions of this article will be made available by the authors, without undue reservation, to any qualified researcher.

## Ethics Statement

The studies involving human participants were reviewed and approved by the Comité de Ética de la Investigación de la Universidad Autónoma de Madrid. The patients/participants provided their written informed consent to participate in this study.

## Author Contributions

ML-S was the principal author and the one who has contributed most to the manuscript presented here, was elaborated the theoretical framework that supports the article, as well as the realization of the different analyses that were presented. DG-D was one of the contributors who have focused on making contributions to the theoretical framework and the final discussions. IM was one of the contributors who focused on making contributions to the statistical analysis and discussions derived from it. All authors contributed to the article and approved the submitted version.

## Conflict of Interest

The authors declare that the research was conducted in the absence of any commercial or financial relationships that could be construed as a potential conflict of interest.
